# Investigation of Chemical Constituents of *Eranthis longistipitata* (Ranunculaceae): Coumarins and Furochromones

**DOI:** 10.3390/ijms23010406

**Published:** 2021-12-30

**Authors:** Andrey S. Erst, Alexander A. Chernonosov, Natalia V. Petrova, Maxim S. Kulikovskiy, Svetlana Yu. Maltseva, Wei Wang, Vera A. Kostikova

**Affiliations:** 1 Central Siberian Botanical Garden, Siberian Branch of Russian Academy of Sciences, CSBG SB RUS, 630090 Novosibirsk, Russia; 2 Laboratory Herbarium (TK), Tomsk State University, 634050 Tomsk, Russia; 3 Institute of Chemical Biology and Fundamental Medicine, Siberian Branch of Russian Academy of Sciences, ICBFM SB RAS, 630090 Novosibirsk, Russia; alexander.chernonosov@niboch.nsc.ru; 4 Komarov Botanical Institute, Russian Academy of Sciences, BIN RAS, 197376 St. Petersburg, Russia; npetrova@binran.ru; 5 K.A. Timiryazev Institute of Plant Physiology RAS, IPP RAS, 127276 Moscow, Russia; max-kulikovsky@yandex.ru (M.S.K.); svetadm32@gmail.com (S.Y.M.); 6 Institute of Botany, Chinese Academy of Sciences, IB CAS, Beijing 100093, China; wangwei1127@ibcas.ac.cn

**Keywords:** Ranunculaceae, *Eranthis longistipitata*, LC-HRMS, chemical composition, coumarin, furochromone, fatty acid, extract

## Abstract

Aqueous-ethanol extracts (70%) from the leaves of *Eranthis longistipitata* Regel. (Ranunculaceae Juss.)—collected from natural populations of Kyrgyzstan—were studied by liquid chromatography with high-resolution mass spectrometry (LC-HRMS). There was no variation of the metabolic profiles among plants that were collected from different populations. More than 160 compounds were found in the leaves, of which 72 were identified to the class level and 58 to the individual-compound level. The class of flavonoids proved to be the most widely represented (19 compounds), including six aglycones [quercetin, kaempferol, aromadendrin, 6-methoxytaxifolin, phloretin, and (+)-catechin] and mono- and diglycosides (the other 13 compounds). In the analyzed samples of *E. longistipitata*, 14 fatty acid–related compounds were identified, but coumarins and furochromones that were found in *E. longistipitata* were the most interesting result; furochromones khelloside, khellin, visnagin, and cimifugin were found in *E. longistipitata* for the first time. Coumarins 5,7-dihydroxy-4-methylcoumarin, scoparone, fraxetin, and luvangetin and furochromones methoxsalen, 5-*O*-methylvisammioside, and visamminol-3′-O-glucoside were detected for the first time in the genus *Eranthis* Salisb. For all the above compounds, the structural formulas are given. Furthermore, detailed information (with structural formulas) is provided on the diversity of chromones and furochromones in other representatives of *Eranthis*. The presence of chromones in plants of the genus *Eranthis* confirms its closeness to the genus *Actaea* L. because chromones are synthesized by normal physiological processes only in these members of the Ranunculaceae family.

## 1. Introduction

Plant extracts are most often multicomponent mixtures and, therefore, it is difficult to analyze individual substances there. Liquid chromatography coupled with mass spectrometry (LC-MS) is widely used as a powerful tool for the analysis of substances in plant extracts. Recently, with the development of various analytical methods, such as high-resolution mass spectrometry (HRMS), it became possible to quickly and selectively identify components in extracts. Due to its high accuracy and analytical sensitivity, LC-HRMS greatly facilitates the identification of known and unknown compounds in extracts [1,2]. Furthermore, this method does not require a large amount of plant material for the assay; this feature is undoubtedly suitable for studying plants with a small geographic range and limited occurrence in nature. Such plants include almost all members of the genus *Eranthis* Salisb.

The genus *Eranthis* belongs to Ranunculaceae Juss. Tribe Cimicifugeae Torr. & A. Grey [3]. This genus consists of 10 to 13 early flowering herbaceous perennial species that are distributed across Southern Europe and Western, Central, and temperate Asia [4,5,6,7]. Species of this genus have rarely been the subject of phytochemical research. Examination of the literature indicates that triterpene glycosides and saponins have been studied (along with their biological activity) in *E. cilicica* Schott & Kotschy [8,9]. The set of flavonoids has been investigated in *E. sibirica* DC., *E. stellata* Maxim., *E. longistipitata* Regel., and *E. tanhoensis* Erst [10,11]. Nonetheless, the chromones that were found in *E. cilicica*, *E. pinnatifida* Maxim., and *E. hyemalis* (L.) Salisb. Are the most intriguing finding [12,13,14,15,16], and the same is true for another family of benzo-α-pyrone derivatives: coumarins. In contrast to other classes of *Eranthis* chemical constituents, which are widespread in the plant kingdom and have been studied sufficiently, the limited occurrence of chromones and coumarins and their pharmacological activity are of much interest.

The aim of this work was to identify numerous chemical compounds in extracts from *E. longistipitata* leaves by LC-HRMS for subsequent chemotaxonomic, phytochemical, and pharmacological studies.

## 2. Results

**Figure 1:**
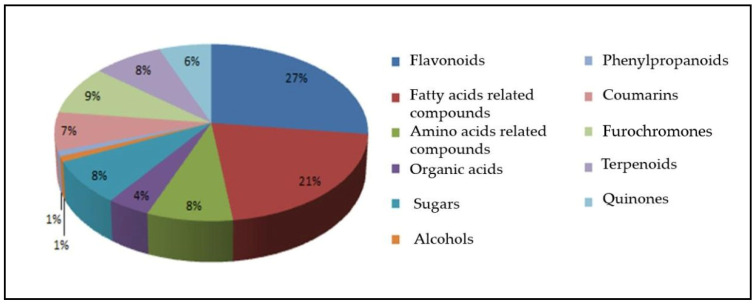
Numbers (as % of total) of bioactive compounds (by class) that were detected in *E. longistipitata* leaves.

Compounds belonging to such classes as amino acids, flavonoids, and fatty and organic acids—as well as their derivatives, alcohols, sugars, coumarins, furochromones, and others—were analyzed by mass spectrometry (LC-HRMS) in *E. longistipitata* leaves (in 70% aqueous-ethanol extracts) from natural populations growing in Kyrgyzstan (Table 1 and Figure 1). For identification of compounds, the mass spectrometry (MS) data acquired in negative and positive electrospray ionization (ESI^−^/ESI^+^) modes were compared with information in the mzCloud database. There was a ≤99.9% match of the mass spectra between the compounds obtained by us and compounds from the mzCloud database (Table 1). A more detailed procedure for identifying substances is described in Ref. [11].

## 3. Discussion

In the raw data on metabolites from *E. longistipitata* leaves, more than 160 compounds were found, of which, 72 were identified to the class level (Figure 1) and 58 to the individual-compound level (Table 1). After a comparison of *E. longistipitata* metabolomes among different populations, it turned out that these profiles of metabolites do not differ and are characterized by the presence of such classes of compounds as flavonoids, fatty acid–related compounds, amino acid–related compounds, organic acids, sugars, alcohols, phenylpropanoids, coumarins, furochromones, terpenoids, and quinones. Among the classes of substances that were identified in the leaves of *E. longistipitata*, the highest diversity was documented for flavonoids (27% of all compounds) and fatty acid–related compounds (21%). Furochromones showed lower diversity (9%), as did terpenoids, amino acid–related compounds, and sugars (8% each), coumarins (7%)**,** and quinones (6%). Other classes of compounds were not so diverse in the extracts of this plant: organic acids (4% of all compounds), alcohols (1%), and phenylpropanoids (1%). We failed to find metabolomic data on *Eranthis* representatives, including *E. longistipitata*, in the literature.

### 3.1. Flavonoids

The class of flavonoids proved to be most widely represented (19 compounds), among which there were only six aglycones [quercetin, kaempferol, aromadendrin, 6-methoxytaxifolin, phloretin, and (+)-catechin] and the remaining flavonoids were mono- and diglycosides, whose sugar moiety consisted of glucose, galactose, arabinose, or other sugars. We reported about the specific features of *E. longistipitata* flavonoids in more detail in our previous article [11]. In addition, here we were able to identify a quercetin diglycoside: quercetin-6-*O*-β-d-xylopyranosyl-β-d-glucopyranoside (Table 1).

### 3.2. Fatty-Acid–Related Compounds

In the analyzed extracts of *E. longistipitata* leaves, 14 fatty acid–related compounds were identified (Table 1). Mostly, these are long-chain fatty acids, octadecanoids with hydroxyl substituents, oxylipins, and hydroxypolyunsaturated fatty acids. The identified fatty acids play an important role in the metabolism of plant organisms and have various types of biological activity [17,18]. For example, 9-oxo-ODA, by acting on peroxisome proliferator-activated receptor, inhibits cellular triglyceride accumulation in hepatocytes and can be used against disorders of lipid metabolism [19]. 12-Oxo-phytodienoic acid is affiliated with jasmonates: oxylipins that serve as key signaling compounds in immunity, germination, and development of plants [20,21]. 12-Oxo-phytodienoic acid plays an important part in plant resistance to water-deficit conditions by regulating stomatal aperture [22]; its anti-inflammatory activity is documented [23]. Previously, the fatty acid profile was characterized only in the seeds of *E. hyemalis*, even though the fatty acid profiles differ among individual genera of the Ranunculaceae family and may have taxonomic significance [24].

### 3.3. Coumarins

**Figure 2:**
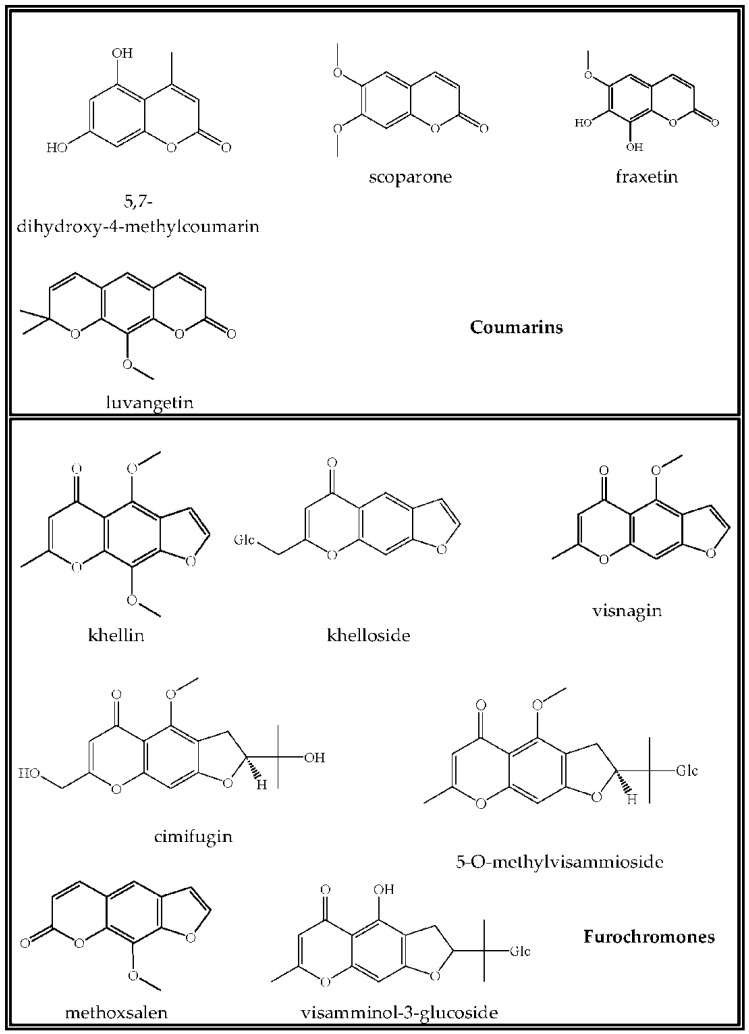
Structures of coumarins and furochromones from *E. longistipitata*.

Natural compounds that are based on 5,6-benzo-α-pyrone are known as coumarins. Investigation into the chemistry of coumarins from the Ranunculaceae family began in the last century and continues to develop rapidly at present. The stimuli for these research advances are, on the one hand, the high variety of natural coumarins, and on the other hand, the practical value of many of these compounds as biologically-active substances. In *E. longistipitata*, four substances that were related to coumarins were identified here: 5,7-dihydroxy-4-methylcoumarin, scoparone, fraxetin, and luvangetin (Table 1, Figure 2). The first three compounds consist of a coumarin molecule with the following substituents: hydroxyl groups at positions C-5 and C-7 and a methyl group at C-4 in 5,7-dihydroxy-4-methylcoumarin; there are methoxy groups at positions C-6 and C-7 in scoparone, whereas fraxetin also contains a methoxy group at the C-6 position and two hydroxyl groups at positions C-7 and C-8. All these simple coumarins are widespread in the plant world and have various biological activities [25,26]. Due to the high biological activity and low toxicity of coumarins, the attention of researchers in recent years was focused on the antitumor effects of these substances. Coumarins are used in the treatment of renal cell carcinoma, leukemia, and prostate cancer and have the ability to counteract the adverse effects of radiotherapy [27].

Via the addition of a pyran ring, simple coumarins can be converted to pyranocoumarins, such as luvangetin which was found in *E. longistipitata* here. Luvangetin is a linear pyranocoumarin consisting of a six-membered ring that is linked to the benzene site of a benzopyran ring with a double bond at the C-3′(4′) position and a methoxyl group at C-8. Luvangetin possesses impressive biological properties such as antiulcer [28] and antibacterial [29] effects and antifungal activity against *Fusarium graminearum* Schwabe, *Rhizoctonia solani* J.G. Kuhn, and *Pyricularia* oryzae Cavara [30]. The gastroprotective effect of luvangetin is not fully characterized because at a concentration of 1–10 μg/mL, it does not induce additional production of prostaglandins as previously thought; luvangetin probably stimulates other protective actions of the mucous membrane [31].

### 3.4. Chromones

Chromones, a group of secondary metabolites that are based on 4H-1-benzopyran-4-one, are considered common among a relatively limited number of plant species that are affiliated with families Ranunculaceae, Apiaceae Lindl., and Fabaceae Lindl., and some others [32]. Among the Ranunculaceae species, chromones have been found only in species of *Eranthis* [12,13,14,15,16,33] and of *Actaea* L. (sin. *Cimicifuga* Wermisch.) [34,35,36,37,38]. In terms of their structure, chromones are close to coumarins and flavonoids but are much less common in nature. Slightly more than 50 chromones are known, and their structural diversity has led to their tentative categorization into simple chromones and benzo-, pyrano-, and furo-chromones. Previously, in the *Eranthis* species that have been studied, compounds from the families of simple chromones and furochromones have been found (Table 2).

In the series of substances **1**–**6**, substituents vary at positions C-2 and C-12. At C-2, it is either a methyl group or a hydroxymethyl group whereas at the C-12 position: mono- and diglycosides. In compounds **7***–***12**, the oxepin ring is open and the 4′-hydroxy-3′-methylbut-2′-enyl group is fixed at the C-8 position. D-Glucose can be present at the C-7 position (as in compounds **9** and **10**) or at the C-4′ position (as in compounds **11** and **12**). Compounds **13** and **14** are close in structure to substances **1**–**6**, and the differences are as follows: the double bond in the oxepine ring is shifted from C-9(10) to C-10(11), respectively, and C-9 carries not only the oxymethylene group with various substituents but also a hydroxyl group.

Most often, the presence of simple chromones has been registered in the underground part of *Eranthis* species, whereas furochromones have been identified in both the underground and aboveground parts (Table 2).

The furochromones that have been registered in *Eranthis* species are represented by cimifugin (**15**) and two of its derivatives: cimicifugin β-d-glucopyranoside (**16**) and norcimifugin (**17**). Khellin (**19**) is a furanochromone derivative in which there are methoxy groups at positions C-4 and C-9 and a methyl group at the C-7 position (Table 2). Visnagin (**18**) is based on the furanochromone framework too but has substituents at positions C-4 and C-7 (methoxy and methyl groups, respectively), but there are no substituents at the C-9 position. Khellol (**20**) is an aglycone of khellol glucoside (**21**), whose sugar moiety is attached at the C-7 position. The next two compounds that were previously found in the leaves and stems of *E. pinnatifida* differ from each other by the presence of a hydroxymethyl group at the C-9 position [in norammiol (**23**)] or its absence [in norkhellol (**22**)] [13]. The furochromone that was identified in tubers of *E. cilicica* (**24**) is a diglucoside [16].

In our extracts of *E. longistipitata*, the class of chromones is represented by seven compounds belonging to the family of furochromones (Table 1, Figure 2). The latter are formed via condensation of simple chromones with a furan ring at positions C-6 and C-7. Cimifugin (**15**) has been detected previously in the leaves and stems of *E. pinnatifida* and in the tubers of *E. cilicica*; visnagin (**18**), khellin (**19**)*,* and khellol glucoside (**21**) have been found in *E. hyemalis*, whereas khellol (**20**) in the leaves and stems of *E. pinnatifida* (Table 2). In our study, methoxsalen, 5-*O*-methylvisammioside, and visamminol-3′-O-glucoside were found in *Eranthis* for the first time. Methoxsalen is a naturally occurring analog of psoralen and is found in various species of Rutaceae Juss, Fabaceae, and Apiaceae [39,40,41]. Earlier, 5-O-methylvisammioside was isolated from the underground part (radix) of *Saposhnikovia divaricata* (Turcz. ex Ledeb.) Schischk. (Apiaceae) [42].

The pharmacological activity of furochromones and their limited occurrence are of interest. Chromone derivatives possess anti-inflammatory, antiviral, and antitumor activities and are employed as antioxidants. In addition, due to their photochemical properties, they can serve as fluorescent labels in biochemical experiments and clinical practice. Chromones, with electron-withdrawing substituents at the C-3 position, form the basis for the synthesis of various heterocyclic compounds [43,44,45]. The first chromone to be used in clinical practice was khellin, which is isolated from the seeds of *Ammi visnaga* (L.) Lam. [45]. Khellin and visnagin can serve as bioherbicides [46] and an anti-inflammatory activity of 5-O-methylvisammioside has been reported [47], whereas cimifugin effectively inhibits allergic inflammatory reactions [48]. Some chromones that were isolated from *Eranthis* exert antioxidant action on the superoxide anion [16].

According to comprehensive molecular and morphological analyses, the genus *Eranthis* is affiliated with Ranunculaceae tribe Cimicifugeae, which includes genera *Actaea* (incl. *Cimicifuga* and *Souliea* Franch.), *Anemonopsis* Siebold & Zucc., *Beesia* Balf.f. & W.W.Sm., and *Eranthis* [3]. On the basis of morphological similarity with the genus *Helleborus* L., some investigators have assigned *Eranthis* to the tribe Helleboreae DC., subtribe Helleborinae Jensen [49,50,51]. On the other hand, ongoing research in the field of *Helleborus* phytochemistry indicates that a distinctive feature of this genus is the accumulation of substances from the class of cardioactive glycosides: cardenolides and bufadienolides [52], which have not been found in *Eranthis* species. In phytochemical characteristics, *Eranthis* is closer to the genus *Actaea* because chromones are synthesized in these genera via normal physiological processes [53]. Our detection of furochromones in *E. longistipitata* is another proof of the close relationship between *Eranthis* and the genus *Actaea*.

## 4. Materials and Methods

### 4.1. Plant Material and Preparation of the Extract

The leaves of *E. longistipitata* were collected during the flowering–fruiting period in 2019 (Table 3). The plant material was dried by means of silica gel. The leaves were ground up to obtain a homogeneous powder. The set of compounds was studied in 70% aqueous-ethanol extracts of the leaves; these extracts were prepared at 72 C in a WB-4MS water bath (Biosan, Riga, Latvia). A certain portion (0.200 g) of the crushed air-dried material was extracted twice: first, with 30 mL for 30 min, and then, with 20 mL for 20 min. “Yellow ribbon” filter paper (LLC Melior XXI, Reutov, Russia) was used to filter extracts. After filtration, the residue in the flask and on the filter was washed with 5 mL of 70% ethyl alcohol. The mixed extract was then concentrated in porcelain cups down to 5 mL. Before the analysis, the solutions were filtered and stored at 4 °C.

### 4.2. MS Settings and the Spectral Library

An aqueous-ethanol extract (1 mL) was diluted with double-distilled water to 5 mL and passed through a Diapak C16 concentrating cartridge (ZAO BioKhimMak, Moscow, Russia). The substances were washed off the cartridge with a small amount (3 mL) of 70% ethanol and then with 2 mL of 96% ethanol. The combined eluate was passed through a membrane filter with a pore diameter of 0.45 μm (Restek, Shanghai, China).

LC-HRMS was carried out at the Core Facility of Mass-Spectrometric Analysis at the Institute of Chemical Biology and Fundamental Medicine SB RAS (Novosibirsk, Russia). An Ultimate 3000 liquid chromatograph (Thermo Fisher Scientific, San Jose, CA, USA) that was coupled with a Q Exactive HF mass spectrometer (Thermo Fisher Scientific) was utilized to determine metabolomic profiles of *E. longistipitata* leaves. The chromatographic separation was attained at a 0.4 mL/min flow rate on a Zorbax Eclipse XDB-C18 reversed-phase column (150 × 3.0 mm, 5 μm, Agilent Technologies, Santa Clara, CA, USA) thermostatted at 40 °C. The mobile phase was composed of 0.1% aqueous formic acid (eluent A) and acetonitrile (eluent B). The elution gradient was implemented as follows: from 5% to 70% B for 40 min, followed by an increase to 90% B for 8 min, a decrease to 5% B for 5 min, and re-equilibration under the initial conditions for 7 min.

The settings of the ESI source were as follows: electrospray voltage: 3.2 kV in the negative mode and 4.2 kV in the positive mode; capillary temperature: 320 °C; and the S lens RF level: 50. The data were obtained by full-scan data-dependent acquisition (FS-dd-MS2) in the positive and negative modes at a resolving power of 45,000 full-width at half maximum (FWHM) at *m*/*z* 200. The following settings of the mass spectrometer were employed: the scan range, *m*/*z* 80–1200; automatic gain control (AGC), 3e6; injection time, 100 ms; and the isolation window, *m*/*z* 2.0. The normalized collision energy for the fragmentation of molecular ions was set to 40 eV. A targeted MS/MS (dd-MS2) analysis was performed in both the positive and negative modes at 15,000 FWHM (at *m*/*z* 200). AGC for dd-MS2 was set to 1 × 10^5^, with an injection time of 50 ms and a loop count of 5. In the section of dd settings, the AGC target was programmed at 8 × 10^3^, and the maximum injection time was set to 100 ms. The data were analyzed using Xcalibur 4.0 and Compound Discoverer 3.1 software (Thermo Fisher Scientific). All the samples, including the blank samples, were assayed in triplicate.

All the samples were processed in Compound Discoverer 3.1 via a common workflow called “Environmental Unknown ID w Online and Local Database Searches” (Figure S1 in Supplementary Material). A mass tolerance of 5 ppm was applied to all nodes. Several databases, i.e., KEGG (https://www.genome.jp/kegg/; last accessed on 10 March 2021), MassBank (https://massbank.eu/MassBank/; last accessed on 10 March 2021), PlantCyc (https://plantcyc.org/; last accessed on 10 March 2021), and Planta Piloto de Quimica Fina Universidad de Alcala (http://www.cqab.eu/index.php/en/; last accessed on 10 March 2021), were chosen in ChemSpider.

The chemical constituents were identified on the basis of both accurate mass and fragment mass “fingerprint” spectra via searches against the spectra of compounds that were available in the mzCloud database (https://www.mzcloud.org; last accessed on 10 March 2021). If compounds were absent in mzCloud, they were tentatively identified using a ChemSpider search. According to the workflow, the masses that were extracted from the chromatograms were aligned and filtered to remove (i) the background compounds that were present in the blank sample, (ii) substances that failed to become fragmented, (iii) compounds’ masses that were absent in the databases, and (iv) signals with low intensity.

All the samples, including the blank samples, which consisted of the pure solvent, were analyzed as two biological replicates with three technical replicates per treatment group.

### 4.3. Chemicals

All chemicals were of MS or analytical grade. The chemical reference standards of quercetin and kaempferol were purchased from Sigma-Aldrich (Taufkirchen, Germany). Rutin and chemical reference standards of hyperoside were purchased from Fluka Chemie AG (Buchs, Switzerland).

## 5. Conclusions

Data on the overall metabolite profile of *E. longistipitata* leaves were obtained by LC-HRMS for the first time. This method requires only a small amount of plant material and, therefore, is certainly suitable for the research on endemic plants of the genus *Eranthis*. A comparison of samples of *E. longistipitata* among different natural populations revealed no differences in the metabolome among the studied samples. Next, we more closely examined flavonoids in the metabolite profile (19 compounds). Additionally, coumarins (four compounds), amino acids (six), fatty (14) and organic acids (three), sugars (three), furochromones (seven), and other compounds were identified in the leaves. Flavonoids and fatty acids manifested the highest diversity; however, coumarins and furochromones that were found in *E. longistipitata* are more intriguing and are relatively rare in the plant kingdom. The proposed fine-tuned technique for the identification of substances in *E. longistipitata* leaves will help investigators to conduct further phytochemical and chemotaxonomic research on plant species from the genus *Eranthis*.

## Figures and Tables

**Table 1:** Chemical constituents identified in *E. longistipitata* leaves (aqueous-ethanol extracts) using LC-HRMS, mzCloud, and ChemSpider.

Identified Compounds	t_R_ (min)	Calculated Mass	Measured Mass	Delta Mass [Da]	Delta Mass [ppm]	MzCloud Score	Mode
Flavonoids							
Quercetin *	11.29	302.0426	302.0423	−0.00035	−1.15	99.9	Positive
Isoquercitrin (quercetin-3-*O*-β-d-glucoside)	13.01	464.0954	464.0958	0.00035	0.75	99.2	Negative
Hyperoside * (quercetin 3-*O-*β-d-galactoside)	10.95	464.0954	464.0952	−0.00024	−0.51	98.2	Positive
Reynoutrin (quercetin-3-*O*-β-d-xylopyranoside)	10.95	434.0849	434.0846	−0.00031	−0.72	98.7	Positive
Quercetin-6-O-β-d- xylopyranosyl-β-d- glucopyranoside	11.65	596.13773	596.13714	−0.0006	−1	98.1	Negative
Quercetin 3-sambubioside (quercetin-3-*O*-[β-d-xylosyl- (1→2)-β-d-glucoside])	11.66	596.1377	596.1371	−0.00060	−1.00	98.1	Negative
Peltatoside (quercetin-3-(6-*O*-α-l- arabinopyranosyl)-β-d- glucopyranoside))	10.13	596.1377	596.1368	−0.00084	−1.42	-	Positive
Rutin * (quercetin 3-*O*-β-d- rutinoside)	12.48	610.1533	610.1524	−0.00089	−1.46	98.9	Positive
Kaempferol *	11.90	286.0477	286.0475	−0.00023	−0.80	99.0	Positive
Juglalin (kaempferol 3-*O*-α-l- arabinopyranoside)	11.90	418.0900	418.0896	−0.00033	−0.79	79.8	Positive
Trifolin (kaempferol-3-*O*-β-d-galactoside)	11.90	448.1005	448.1003	−0.00024	−0.54	98.5	Positive
Carlinoside (luteolin 6-*C*-β-d- glucopyranoside-8-*C*-α-l- arabinopyranoside)	12.56	580.1428	580.1425	−0.00023	−0.40	-	Negative
Cianidanol [(+)-catechin]	16.53	290.0790	290.0789	−0.00003	−0.11	-	Positive
Auriculoside (7,3′,5′-trihydroxy-4′-methoxyflavan-3′-glucoside)	19.78	450.1526	450.1521	−0.00045	−0.99	-	Positive
6-Methoxytaxifolin	14.55	334.0688	334.0690	0.00016	0.48	-	Negative
Aromadendrin ((+)-dihydrokaempferol)	21.50	288.0633	288.0633	−0.00003	−0.11	-	Positive
Aspalathin	15.45	452.1318	452.1317	−0.00014	−0.32	-	Positive
Phloridzin (phloretin-2′-*O*-β-glucoside)	16.23	436.1369	436.1368	−0.00013	−0.30	-	Positive
Phloretin (dihydroxy naringenin)	20.80	274.0841	274.0839	−0.00019	−0.69	-	Positive
Fatty acids-related compounds							
12-Oxo-phytodienoic acid	36.84	292.2038	292.2037	−0.00013	−0.43	87.3	Positive
15-OxoEDE (15-Oxo-11Z,13E-eicosadienoic acid)	47.47	322.2507	322.2508	0.00006	0.2	91.7	Positive
9-oxo-ODA (9-Oxo-10(E),12(E)-octadecadienoic acid)	39.22	294.2194	294.2194	−0.00006	−0.21	95.3	Positive
9S,13R-12-Oxo-phytodienoic acid	36.47	292.2038	292.2037	−0.00009	−0.3	87.2	Positive
Linolenic acid ethyl ester	46.47	306.2558	306.2558	−0.00008	−0.28	98.3	Positive
Palmitoleic Acid	45.38	254.2245	254.2245	−0.00006	−0.24	86.7	Positive
α-Eleostearic acid	49.77	278.2245	278.2244	−0.00017	−0.62	99.1	Positive
α-Linolenic acid	45.50	278.2245	278.2244	−0.00012	−0.45	99.2	Positive
(+/−)13-HODE (13-hydroxyoctadecadienoic acid)	38.17	296.2351	296.2352	0.0001	0.35	92.8	Negative
(15Z)-9,12,13-Trihydroxy-15- octadecenoic acid	24.63	330.2406	330.2408	0.00019	0.57	68.5	Negative
13(S)-HOTrE (13-OH-9Z,11E,15Z-octadecatrienoic acid)	36.23	294.2194	294.2195	0.00009	0.29	92.1	Negative
16-Hydroxyhexadecanoic acid	44.84	272.2351	272.2353	0.0002	0.73	89.5	Negative
Corchorifatty acid F	23.35	328.2249	328.2249	0.00002	0.06	93	Negative
Pinolenic acid	45.76	278.2245	278.2245	−0.0006	−0.21	94.5	Negative
Amino acid-related compounds							
d-(+)-Pyroglutamic Acid	1.56	129.0425	129.0428	0.00021	1.63	98.2	Positive
d-(+)-Tryptophan	6.96	204.0898	204.0898	−0.0003	−0.13	99.2	Positive
Isoleucine	1.93	131.0946	131.0947	0.00014	1.10	99.2	Positive
l-Phenylalanine	4.45	165.0789	165.0790	0.00003	0.19	98.6	Positive
L-Tyrosine	1.93	181.0738	181.0740	0.00015	0.81	98.4	Positive
d-(-)-Glutamine	1.56	146.0691	146.0684	−0.00073	−4.99	66.9	Negative
Organic acids							
Citric acid	2.00	192.027	192.0264	−0.00056	−2.93	99.6	Negative
d-α-Hydroxyglutaric acid	1.99	148.0371	148.0364	−0.00071	−4.81	68.8	Negative
Gluconic acid	1.61	196.0583	196.0577	−0.00057	−2.89	99	Negative
Sugars							
α-Lactose	1.61	342.1162	342.1160	−0.00014	−0.41	81.8	Positive
d-(+)-Galactose	1.61	180.0633	180.0629	−0.00047	−2.61	60.5	Negative
α.α-Trehalose	1.64	342.1162	342.1160	−0.00019	−0.55	98.9	Negative
Phenylpropanoid							
6-Gingerol	28.34	294.1831	294.1830	−0.00004	−0.14	81.8	Positive
Coumarins							
5,7-Dihydroxy-4-methylcoumarin	17.32	192.0422	192.0423	0.00007	0.35	99.9	Positive
Scoparone (6,7-dimethoxycoumarin)	24.79	206.0579	206.0579	0.00002	0.12	98.8	Positive
Fraxetin (7,8-dihydroxy-6-methoxycoumarin)	11.39	208.0371	208.0371	−0.00005	−0.24	96.1	Positive
Luvangetin (10-methoxy-2,2-dimethylpyrano[3,2-g]chromen-8-one)	32.62	258.0892	258.0891	−0.00005	−0.19	93.6	Positive
Furochromones							
Khelloside (7-hydroxymethyl-4-methoxy-5H-furo[3,2-g](1)benzopyran-5-one glucoside)	12.02	408.1056	408.1048	−0.00077	−1.9	99.5	Positive
Khellin (4,9-dimethoxy-7-methyl-5H-furo[3,2-g]chromen-5-one)	21.003	260.0684	260.0680	−0.00039	−1.5	99.8	Positive
Visnagin (4-methoxy-7-methyl-5H-furo[3,2-g]chromen-5-one)	21.138	230.0579	230.0576	−0.00023	−0.98	99.3	Positive
Cimifugin ((2S)-7-(hydroxymethyl)-2-(1-hydroxy-1-methyl-ethyl)-4-methoxy-2,3-dihydrofuro[3,2-g]chromen-5-one)	13.544	306.1103	306.1097	−0.00061	−1.98	95.1	Positive
Methoxsalen (9-methoxyfuro[3,2-g]chromen-7-one)	27.757	216.0422	216.0422	0	0.02	99.3	Positive
5-O-Methylvisammioside (4-O-β-d-glucosyl-5-O-methylvisamminol)	14.538	452.1682	452.1677	−0.00048	−1.05	99.1	Positive
Visamminol-3′-O-glucoside (4-hydroxy-2-(2-hydroxypropan-2-yl)-methyl-2,3-dihydrofuro[3,2-g] chromen-5-one)	16.894	438.1526	438.1523	−0.00022	−0.5	98.2	Positive

* Identification confirmed with the help of standards; the en dash means that ChemSpider was used without mzCloud.

**Table 2:** Chromones that were previously found in *Eranthis* species.

ID	Compound (ID)	Structure	Sourse	Ref.
	CHROMONES			
	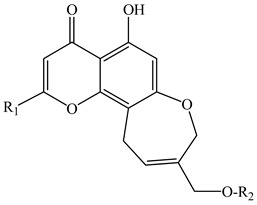			
**1**	8,11-dihydro-5-hydroxy-2,9-dihydroxymethyl-4H-pyrano[2,3-g][1]benzoxepin-4-one	R_1_ = O-CH_2_-OH R_2_ = H	*E. cilicica* (tubers)	[16]
**2**	Eranthin (5-hydroxy-9-hydroxymethyl-2-methyl-8,11-dihydro-4H-pyrano[2,3-g][1]benzoxepin-4-one)	R_1_ = CH_3_ R_2_ = H	*E. hyemalis* (rhizome)	[14]
**3**	Eranthin-β-d-glucoside (9-{[(β-d-glucopyranosvl)oxy]methyl}-8,11-dihydro-5-hydroxy-2-methyl-4*H*-pyrano[2,3-*g*][1]benzoxepin-4-one)	R_1_ = CH_3_ R_2_ = β-d-glucopyranosyl	*E. hyemalis* (rhizome) *E*. *hyemalis* (tubers)	[14,15]
**4**	Eranthin 9-β-d-glucopyranosyl-(1→6)-β-d-glucopyranoside	R_1_ = CH_3_ R_2_ = glucopyranosyl-(1→6)-glucopyranoside	*E. cilicica* (tubers) *E. hyemalis* (tubers)	[15,16]
**5**	Eranthin β-d-gentiobioside (9-{[(β-d-gentiobiosyl)oxy]methyl}-8,11-dihydro-5-hydroxy-2-methyl-4*H*-pyrano[2,3-*g*][1]benzoxepin-4-one)	R_1_ = CH_3_ R_2_ = β-D-gentiobiosyl	*E. hyemalis* (tubers)	[15]
**6**	2-C-Hydroxyeranthin β-d-glucopyranoside (9-{[(β-d-glucopyranosyl)oxy]methyl}-8,11-dihydro-5-hydroxy-2-(hydroxymethyl-4*H*-pyrano[2,3-*g*][1]benzoxepin-4-one)	R_1_ = CH_2_-OH R_2_ = β-d-glucopyranosyl	*E. hyemalis* (tubers)	[15]
	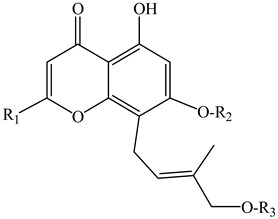			
**7**	5,7-dihydroxy-8-[(2E)-4-hydroxy-3-methylbut-2-enyl]-2-methyl-4H-1-benzopyran-4-one	R_1_ = CH_3_ R_2_ = R_3_=H	*E. cilicica* (tubers)	[16]
**8**	5,7-dihydroxy-2-hydroxymethyl-8-[(2E)-4-hydroxy-3-methylbut-2-enyl]-4H-1-benzopyran-4-one	R_1_ = CH_2_-OH R_2_ = R_3_=H	*E. cilicica* (tubers)	[16]
**9**	7-[(β-d-glucopyranosyl)oxy]-5-hydroxy-8-[(2E)-4-hydroxy-3-methylbut-2-enyl]-2-methyl-4H-1-benzopyran-4-one	R_1_ = CH_3_ R_2_ = β-d-glucopyranosyl R_3_ = H	*E. cilicica* (tubers)	[16]
**10**	7-[(β-d-glucopyranosyl)oxy]-5-hydroxy-2-hydroxymethyl-8-[(2E)-4-hydroxy-3-methylbut-2-enyl]-4H-1-benzopyran-4-one	R_1_ = CH_2_-OH R_2_ = β-d-glucopyranosyl R_3_ = H	*E. cilicica* (tubers)	[16]
**11**	7,8-Secoeranthin β-d-glucoside (8-{(2*E*)-4-[(β-d-glucopyranosyl)oxy]-3-methylbut-2-enyl}-5,7-dihydroxy-2-methyl-4*H*-1-benzopyran-4-one)	R_1_ = CH_3_ R_2_ = H R_3_ = β-D-glucopyranosyl	*E. hyemalis* (tubers)	[15]
**12**	2-C-Hydroxy-7,8-Secoeranthin β-d-glucoside (8-{(2*E*)-4-[(β-d-glucopyranosyl)oxy]-3-methylbut-2-enyl}-5,7-dihydroxy-2-(hydroxymethyl)-4*H*-1-benzopyran-4-one)	R_1_ = CH_2_-OH R_2_ = H R_3_ = β-D-glucopyranosyl	*E. hyemalis* (tubers)	[15]
	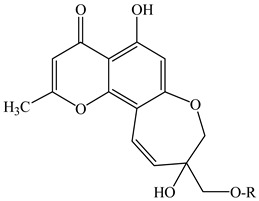			
**13**	9-[(O-β-d-glucopyranosyl-(1→6)-β-d-glucopyranosyl)oxy]methyl-8,11-dihydro-5,9-dihydroxy-2-methyl-4H-pyrano[2,3-g][1]benzoxepin-4-one	R = glucopyranosyl-(1→6)-glucopyranosyl	*E. cilicica* (tubers)	[16]
**14**	8,11-dihydro-5,9-dihydroxy-9-hydroxymethyl-2-methyl-4H-pyrano[2,3-g][1]benzoxepin-4-one	R = H	*E. cilicica* (tubers)	[16]
	FUROCHROMONES			
	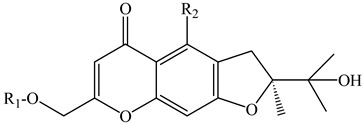			
**15**	Cimifugin (2S)-7-(hydroxymethyl)-2-(2-hydroxypropan-2-yl)-4-methoxy-2,3-dihydrofuro[3,2g]chromen-5-one)	R_1_ = H R_2_ = O-CH_3_	*E. pinnatifida* (leaves, stems) *E. cilicica* (tubers)	[13,16]
**16**	Cimicifugin β-d-glucopyranoside (7-{[(β-d-glucopyranosy1)oxy]methyl}-2,3-dihydro-2-(l-hydroxy-1-methylethyl)-4-methoxy-5*H*-furo[3,2-*g*][1]benzopyran-5-one)	R_1_ = β-D-glucopyranosyl R_2_ = O-CH_3_	*E. hyemalis*	[15]
**17**	Norcimifugin (2S)-4-hydroxy-7-(hydroxymethyl)-2-(2-hydroxypropan-2-yl)-2,3-dihydrofuro[3,2-g]-chromen-5-one)	R_1_ = H R_2_ = OH	*E. pinnatifida* (leaves, stems)	[13]
	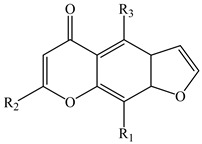			
**18**	Visnagin (4-methoxy-7-methyl-5H-furo[3,2-g]chromen-5-one)	R_1_ = R_2_=H R_3_ = O-CH_3_	*E. hyemalis*	[33]
**19**	Khellin (4,9-dimethoxy-7-methyl-5H-furo[3,2-g]chromen-5-one)	R_1_ = O-CH_3_ R_2_ = H R_3_ = O-CH_3_	*E. hyemalis*	[33]
**20**	Khellol (7-(hydroxymethyl)-4-methoxyfuro[3,2-g]chromen-5-one)	R_1_ = H R_2_ = CH_2_-OH R_3_ = O-CH_3_	*E. pinnatifida* (leaves, stems)	[13]
**21**	Khellol glucoside (khellinin; 2-hydroxymethyl-5-methoxyfuranochrome glucoside)	R_1_ = H R_2_ = CH_2_-β-d-glucopyranoside R_3_ = O-CH_3_	*E. hyemalis* (leaves, flowers)	[12]
**22**	Norkhellol (4-hydroxy-7-(hydroxymethyl)-5H-furo[3,2-g][1]benzopyran-5-one)	R_1_ = H R_2_ = CH_2_-OH R_3_ = OH	*E. pinnatifida* (leaves, stems)	[13]
**23**	Norammiol (4-hydroxy-7(hydroxymethyl)-9-methoxy-5H-furo[3,2-g][1]-benzopyran-5-one)	R_1_ = O-CH_3_ R_2_ = CH_2_-OH R_3_ = OH	*E. pinnatifida* (leaves, stems)	[13]
**24**	7-[(O-β-d-glucopyranosyl-(1→6)-β-d-glucopyranosyl)oxy]methyl-4-hydroxy-5H-furo[3,2-g][1]benzopyran-5-one (**24**)	R_1_ = H R_2_ = CH_2_-O-glucopyranosyl-(1→6)-glucopyranosyl R_3_ = OH	*E. cilicica* (tubers)	[16]

**Table 3:** Sites of collection of the analyzed samples.

Sample No.	Locality; Coordinates	Habitat	Date
1	Kyrgyzstan, Chuya region, Issyk-atinskii district, Karandolot tract; 42°44′22″ N, 74°55’50″ E	foot of the mount	22 March 2019
2	Kyrgyzstan, Talas region, Kara-Buurinskii district, west of Kirovskoe reservoir; 42°37′57″ N, 71°34′47″ E	steppe	26 March 2019

## Data Availability

Not applicable.

## Supplementary Materials

The following are available online at https://www.mdpi.com/article/10.3390/ijms23010406/s1.
