# Supplementary material for: Investigation of Chemical Constituents of Eranthis longistipitata (Ranunculaceae): Coumarins and Furochromones
Source: Int J Mol Sci. 2021 Dec 30;23(1):406. doi: 10.3390/ijms23010406 (PMC8745120; doi:10.3390/ijms23010406)
Supplement: Supplementary file 1 [file ijms-23-00406-s001.zip › ijms-1536969-supplementary.pdf]

Supplementary Material

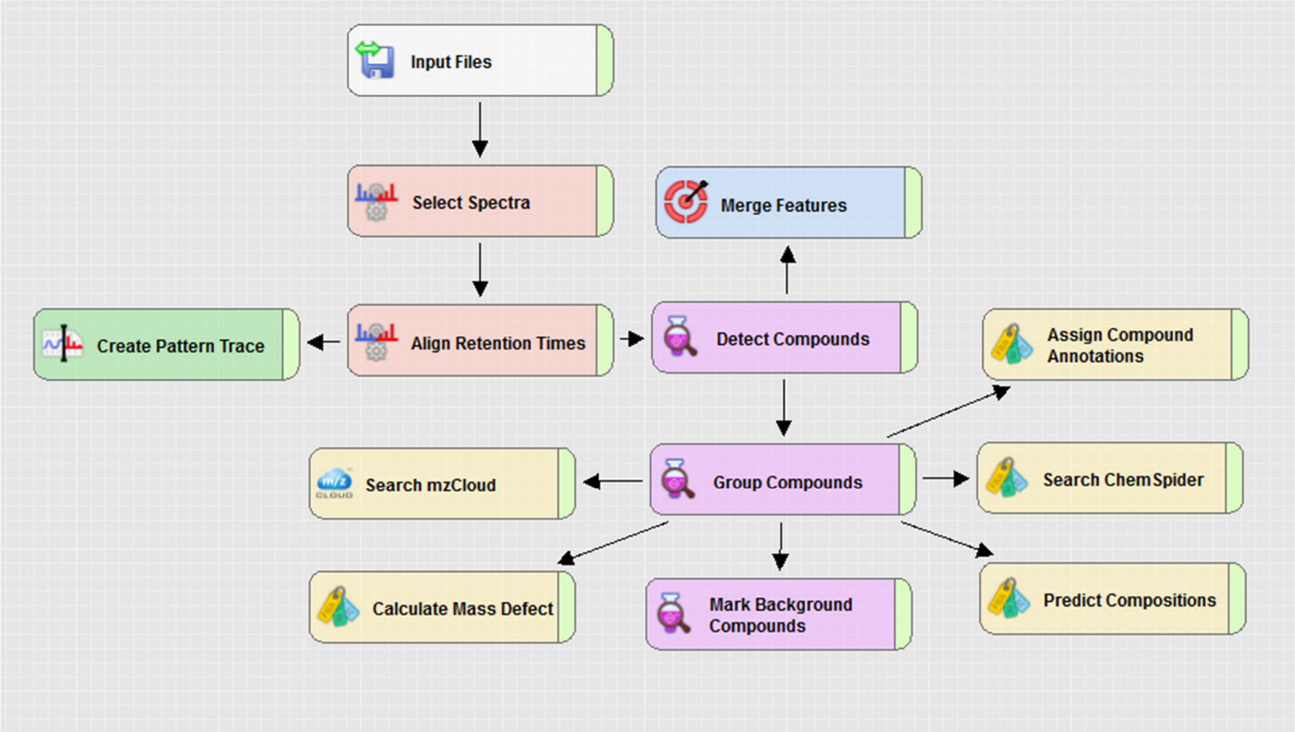

Figure S1. The workflow on Compound Discoverer used for the identification of chemical constituents
